# Success Rate of Nonsurgical Endodontic Treatment of Nonvital Teeth with Variable Periradicular Lesions

**Published:** 2011-08-15

**Authors:** Fariborz Moazami, Safoora Sahebi, Fereshte Sobhnamayan, Abbas Alipour

**Affiliations:** 1. Department of Endodontics, Dental school, Shiraz University of Medical Sciences, Shiraz, Iran.

**Keywords:** Endodontics, Periapical Disease, Retreatment, Root Canal Therapy, Treatment Outcome

## Abstract

**INTRODUCTION:**

Bacterial infection of tooth pulp can progress into periapical diseases. Root canal treatment has been established as the best treatment. In cases of failure, nonsurgical retreatment of teeth is preferred to surgical procedure and extraction.

**MATERIALS AND METHODS:**

In this historical cohort study, 104 permanent teeth with apical lesion were treated during 2002-2008. All teeth showed radiographic evidence of periapical lesion varying in size from 1 to >10mm. A total of 55 teeth were treated with initial root canal treatment and 49 teeth required retreatment. Patients were recalled up to ≈7 years. All radiographs were taken by RSV MAC digital imaging set and long cone technique. The presence/absence of signs and symptoms and periapical index scores (PAI) were used for measuring outcome. Teeth were classified as healed (clinical/radiographic absence of signs and symptoms) or diseased (clinical/radiographic presence of signs and symptoms). The data were statistically analyzed using student t-test and Pearson chi-square or fisher’s exact test.

**RESULTS:**

The rate of complete healing for teeth with initial treatment was 89.7%, and for retreatment group was 85.7%; there was no significant difference. Size of lesions did not significantly affect the treatment outcomes. Success of tooth treatment did not reveal significant correlation with gender and number of roots.

**CONCLUSION:**

Orthograde endodontic treatment/retreatment demonstrates favorable outcomes. Thus, nonsurgical endodontic treatment/retreatment should be considered as the first choice in teeth with large periapical lesion.

## INTRODUCTION

Infection of root canal system occurs subsequent to tooth caries, surgical treatments, and trauma. The microbial flora is commonly mixed, predominantly gram-negative, anaerobic bacterium [1]. The close relation between tooth pulp and periapical region allows passage of bacteria, fungi, and cell components with a path for initiating inflammatory processes in periapical regions and activating resorption in the tissues. These immunopathological mechanisms lead to formation of abscess, granuloma, and periapical cyst [2][3][4]. Ramachandran Nair et al. analyzed 256 periapical lesions histologically and found that 35% were abscesses, 50% were granulomas, while only 15% were cysts; 52% of lesions had an epithelial compartment within their structures [5][6][7]. The incidence of periapical cysts has been reported to be 15-42%. Radiographs cannot distinct periapical radiolucencies as a cyst or granuloma. The two types of periapical cysts are true and pocket cysts. True cyst has lumen with intact epithelial lining which is separate from the root apex; whereas, pocket cyst shows the lumen which is open to the root canal of the infected tooth. True cysts do not probably heal after non-surgical endodontic therapy and usually require surgical procedures [8]. Some clinical studies have shown healing of large periapical lesions following simple endodontic treatments [9][10]. Previously, large periapical lesions were generally managed by surgical excision of cysts after root canal treatment of infected teeth [11]; during recent years, increased knowledge of the morphology and complexity of root canal system has led to development of newer technique, instrument and materials which consequently result in improved endodontic treatment and healing of cyst and a reduced need for periapical surgery [12][13]. Success rate of about 90% is reported for endodontic treatments. Although several studies still believe that treatment for teeth with periapical lesions has lower success rate (20% decrease) [14][15][16].

As there are no studies looking at the healing of endodontic lesions in Iran, the aim of this study was to evaluate the success rate of nonsurgical endodontic treatment/retreatment of teeth with various periapical lesions sizes.

## MATERIALS AND METHODS

For this historical cohort study, 104 permanent teeth of 81 patients were analyzed. All teeth had been endodontically treated during 2002-2008. Patients with endodontic-periodontal lesions, contributory systemic disease, obturation techniques other than lateral condensation, inter-appointment dressings >1 session, and follow-ups <6 months were excluded from this study. An informative form including individual, medical and dental information in addition to detailed records of previous root canal treatments was performed for each patient. Among the included teeth, 41 were single rooted, 7 were double rooted, and the remaining 56 were multiple rooted. Radiographically, all teeth showed periapical lesion with the size between 1mm to >10mm. According to patient records 41 teeth had different sign and symptoms of acute apical periodontitis e.g. pain, tenderness to percussion, localize or diffused swelling and also mobility. The remaining 66 teeth were symptom-free. A total of 55 teeth were root canal treated for the first time and 49 teeth were retreated (failed treatments). Radiographic examination was performed using RSV MAC digital imaging long cone technique. All teeth were treated by one endodontist in one session.

Access cavity was performed and teeth were isolated with rubber dam. Working lengths were determined using appropriate K-files (Mani, Tochigi-ken, Japan). In teeth with previous endodontic treatment, gutta-percha and sealer were removed by hand and rotary instrumentation including Gates-Glidden drills (Mani, Tochigi, Japan), heated plugger, K and H files and also ProTaper rotary system (Dentsply, Maillefer, Ballaigues, Switzerland). If needed chloroform was used as solvent. Working lengths were determined radio-graphically. Subsequently, root canals were instrumented with rotary files. Irrigation was performed frequently with 2.5% sodium hypochlorite (NaOCl). After drying with sterile paper points, canals were obturated with gutta-percha (Ariadent Co., Tehran, Iran) and Tubliseal (Sybron Endo, CA, USA) using cold lateral condensation method. After root canal filling, teeth were restored permanently. Patients were recalled every 4 m for up to 1 yr, and then every 12 m for about 6 yrs.

All radiographs were taken by RSV imaging set and long cone technique with standardized exposure time and no need for processing. The largest diameter of the lesions was measured with RSV imaging software. The presence or absence of signs and symptoms and also PAI scores were used for measuring the outcome.

Teeth were classified as healed when there was clinical absence of signs and symptoms and radiographic PAI score ≤2. Teeth were termed diseased in cases with clinical presence of signs and symptoms or when PAI≥3. Multi-rooted teeth were assigned the highest PAI scores of their roots. Teeth with the absence of any sign or symptoms regardless of PAI score were considered functional. Three trained observers (two endodontist and one radiologist) analyzed radiographs. PAI were assigned to each radiograph. If there was any controversy between observers the two that were similar were chosen.

The data were statistically analyzed using student t-test and Pearson chi-square or fisher’s exact test, where applicable (with a preset probability of P<0.05 and considering of variance equality with Leven test). Experimental results are presented as arithmetic Mean ±SD. Normality of parameters’ distribution was evaluated with one sample Kolmogorov-smirnov test. For the evaluation of non linear association between frequencies of persistent disease state with prognostic factors (independent variables that P-value of association of those with disease status in Univariate analysis was less than 0.2) binary logistic regression was performed.

## RESULTS

Eighty one patient, with 104 teeth (72.1% of teeth were in female patients and 72.1% in males) were evaluated in this historical cohort study with the age ranging from 8-82 years (mean=38.36, SD=13.49). Follow-up time ranged between 4 to 81 month (mean=31.92, SD=21.82). A total of 55 (52.9%) teeth underwent initial treatment and 49 (47.1%) teeth were retreated. Also, 41 teeth (39.4%) were single-rooted and 63 (60.6%) teeth were multi-rooted. Total of 67 (64.4%) teeth had lesion ≤5mm and in the remaining 37 (35.6%) teeth the lesions were ≥5mm. Ninety one teeth (87.5%) were “healed” and the other 13 (12.5%) teeth had persistent disease at the follow-up. Cumulative incidence of healing was 0.875 (95% CI: 0.811, 0.939).

Association between outcome of treatment with demographic and other independent variables were evaluated. Age of patients in healed and diseased groups were 37.57±12.88 and 43.85±16.71 years respectively; which was not statistically significant (P=0.117). Although follow-up time in healed group patients was greater than persistent group patients (32.96±22.11 mon vs. 28.62±20.14 mon), this difference was not statistically significant (P=0.562). Other associations between outcomes of treatment with independent variables are shown in Table 1. There were no remarkable correlation between outcome of treatment with gender and previous treatment status of patients, number of roots and also lesion size.

Multilevel analysis for evaluation of association between outcome of treatment with prognostic variable (root number and age) was evaluated. There were no association between age of patients (OR=1.033; P=0.169) and number of roots (OR=2.092; P=0.293) with treatment outcome.

**Table 1:** Association between outcomes of treatment with independent variables

**Independent variable**		**Outcome of treatment [N (%)]**		***P*-value**
		**Healed (n=91)**	**Diseased (n=13)**	
**Gender**	**Female**	65 (86.7)	10 (13.3)	0.482^a^
	**Male**	26 (89.7)	3 (10.3)	
**Treatment**	**Initial treatment**	49 (89.8)	42 (85.7)	0.602^b^
	**Retreatment**	6 (10.9)	7 (14.3)	
**Root**	**Single-rooted**	38 (92.7)	3 (7.3)	0.162^b^
	**Multi-rooted**	3 (84.1)	10 (15.9)	
**Lesion size**	**≤5mm**	60 (89.6)	7 (10.4)	0.289^a^
	**>5mm**	31 (83.8)	6 (16.2)	

^a^ Fisher exact test

^b^ chi-square test

## DISCUSSION

Unlike other studies that evaluated the success rate in all treated teeth, regardless of periapical lesions, this study focused on the success rate of teeth with periapical lesions. This may explain the difference between the various outcomes.

Several factors may influence endodontic treatment outcome, which are called outcome predictors. Radiographic outcomes have been used to indicate "success" and "failure" of endodontically treated teeth and have been compared with clinical evaluations. Since Goldman et al. demonstrated poor inter- and intra-observer reliability in interpretation of periapical radiographs, and in order to make more reliable criterion PAI was used to describe the status of periapical tissues [17][18].

Unfortunately, methodological problems complicate the comparison of different studies [19]. Several studies have compared the success rate of teeth with and without apical periodontitis (lesions with different size). Most quoted ≈15%-20% lower success rate for teeth with apical periodontitis [20][21][22][23]. This is inconsistent with our study. Peters et al. had a success rate of about 75% in 115 teeth with periapical lesion; 20% lower than the cases without lesions [20]. A further study found a similar pattern with 74% success rates for teeth with apical periodontitis (72 teeth) which was 15% lower than teeth with healthy periapical condition [22]. Farzaneh et al. also showed the success rate of teeth with periapical lesion was 79% in 70 cases which is 14% lower than cases without periapical lesion [21]. The current study shows a success rate of about 87%. Our study may be more reliable than other studies as the experimental procedures were performed by one operator who was a specialist; also factors not assessed in the regression analysis could thus be better controlled (relative to each other) than in retrospective studies with data pooled from a clinic. However, a greater number of teeth were included in this survey, except one other study which may result in better reliability in the treatment outcome.

In the present study, all teeth were treated in one session. Some studies believe that there is no significant difference between one-visit and two-visit endodontic treatments [10][19][23]. Others advocate that using intracanal medicament such as calcium hydroxide between sessions especially in very large periapical lesions is beneficial, as shown in several case reports and studies [23][24][25][26][27][28][29]. Generally, there is a great tendency among practitioners to use calcium hydroxide in canals specifically in those with periapical lesions. In this study we showed high success rate (84%) in cases with large peri-apical lesion without using calcium hydroxide as it is thought that this medicament is not always effective and its action is unreliable [30][31][32][33][34].

The outcome of treatments in this study did not show any correlation with size of lesion. Although there is some evidence to indicate better outcomes for cases with small lesions (≤5mm) compared to those with larger periradicular lesions after either initial treatments [10][23][24][25][35] or retreatments [4]. In some studies, comparable outcomes have been reported for both small and large lesions after initial treatment [23][24][25][35] and retreatment [36]. Soares et al. showed the complete resolution of large periapical lesion after 2-year follow-up [2]; Caroline et al. also showed complete healing of large lesions (10×15mm) after 2yrs [28]. Saatchi demonstrated the 12-month periapical healing of large lesion after using calcium hydroxide as an intracanal dressing [11]. These case reports confirm the high probability of healing of large periapical lesions without periapical surgery, similar to our study. However, Hoskinson et al. suggested that there was nearly an 18% decrease in the probability of success rate with every 1mm increase in the lesion size. He also described the periapical lesion as the most significant factor affecting outcome of treatment which is not in agreement with this study [34]. The better outcome in teeth with smaller lesions that is suggested in this study (though statistically insignificant) is probably due to the greater time required for a large lesion to heal, and the probability of repairing scar tissue in large lesions [10].

Whether RCT was performed as initial treatment or as retreatment did not significantly influence the outcome in this study. This finding is consistent with that of Marending et al. [37] and contrary to other studies like Peters et al., and some other investigators [20][24][38][39][40]. There was an insignificant lower success rate for retreatments, which may be due to treatment complications. Some studies believe in impairment of healing by complications including perforation of the pulp chamber or root, broken instruments that prevent adequate cleaning, and massive extrusion of filling materials [24][35]. Otherwise, the etiology of failure in well-obturated teeth may be more likely related to extraradicular infection, cystic lesions, foreign body reaction, and undiagnosed infractions as the conditions that might not respond to retreatment favorably [41].

This study showed different, but insignificant, outcomes for single-rooted and multi-rooted teeth agreeing with several other studies [14][22][42][43][44]; contrary to study carried out in Toronto phase II and IV [21][45].

The lower outcomes in multi-rooted teeth can be due to the challenge of eliminating root canal infections. However, the difference could be attributed to the use of the tooth as a unit, reflecting the double or triple probability of disease in multi-rooted teeth when assessed according to the worst root (PAI score) [19].

A whole host of studies, with one exception [18], show that gender and age do not significantly affected initial treatment [22][23][24][33][34][35][46] and possibly retreatments [36], concurring with our study.

## CONCLUSION

Nonsurgical endodontic treatment/retreatments are a favored treatment option regardless of the lesion size, previous status of tooth, and age of the patient. Treating teeth with periapical lesions can be performed in one-visit if canals are dry. Further studies with more samples are recommended. Also, longer follow ups may be required for larger lesions and long-term prognosis of endodontic treatments.
